# Sporadic Rift Valley Fever Outbreaks in Humans and Animals in Uganda, October 2017–January 2018

**DOI:** 10.1155/2021/8881191

**Published:** 2021-09-20

**Authors:** Doreen Birungi, Freda Loy Aceng, Lilian Bulage, Innocent Herbert Nkonwa, Bernadette Basuta Mirembe, Claire Biribawa, Denis Okethwangu, Nixon Denis Opio, Fred Monje, David Muwanguzi, Deo Birungi Ndumu, Robert Aruho, Paul Lumu, Julius Lutwama, Benon Kwesiga, Alex Riolexus Ario

**Affiliations:** ^1^Uganda Public Health Fellowship Program, Kampala, Uganda; ^2^Ministry of Agriculture, Animal Industry and Fisheries, Entebbe, Uganda; ^3^Ministry of Health, Kampala, Uganda; ^4^Uganda Wildlife Authority, Kampala, Uganda; ^5^Uganda Virus Research Institute, Entebbe, Uganda

## Abstract

**Introduction:**

Rift Valley fever (RVF) is a mosquito-borne viral zoonosis. The Uganda Ministry of Health received alerts of suspected viral haemorrhagic fever in humans from Kiruhura, Buikwe, Kiboga, and Mityana districts. Laboratory results from Uganda Virus Research Institute indicated that human cases were positive for Rift Valley fever virus (RVFV) by polymerase chain reaction. We investigated to determine the scope of outbreaks, identify exposure factors, and recommend evidence-based control and prevention measures.

**Methods:**

A suspected case was defined as a person with acute fever onset, negative malaria test result, and at least two of the following symptoms: headache, muscle or joint pain, bleeding, and any gastroenteritis symptom (nausea, vomiting, abdominal pain, diarrhoea) in a resident of Kiruhura, Buikwe, Mityana, and Kiboga districts from 1^st^ October 2017 to 30^th^ January 2018. A confirmed case was defined as a suspected case with laboratory confirmation by either detection of RVF nucleic acid by reverse-transcriptase polymerase chain reaction (RT-PCR) or demonstration of serum IgM or IgG antibodies by ELISA. Community case finding was conducted in all affected districts. In-depth interviews were conducted with human cases that were infected with RVF who included herdsmen and slaughterers/meat handlers to identify exposure factors for RVF infection. A total of 24 human and 362 animal blood samples were tested. Animal blood samples were purposively collected from farms that had reported stormy abortions in livestock and unexplained death of animals after a short illness (107 cattle, 83 goats, and 43 sheep). Convenient sampling for the wildlife (10 zebras, 1 topi, and 1 impala) was conducted to investigate infection in animals from Kiruhura, Buikwe, Mityana, and Kiboga districts. Human blood was tested for anti-RVFV IgM and IgG and animal blood for anti-RVFV IgG. Environmental assessments were conducted during the outbreaks in all the affected districts.

**Results:**

Sporadic RVF outbreaks occurred from mid-October 2017 to mid-January 2018 affecting humans, domestic animals, and wildlife. Human cases were reported from Kiruhura, Buikwe, Kiboga, and Mityana districts. Of the 24 human blood samples tested, anti-RVFV IgG was detected in 7 (29%) human samples; 1 human sample had detectable IgM only, and 6 had both IgM and IgG. Three of the seven confirmed human cases died among humans. Results from testing animal blood samples obtained from Kiruhura district indicated that 44% (64/146) cattle, 46% (35/76) goats, and 45% (9/20) sheep tested positive for RVF. Among wildlife, (1/10) zebras, (1/1) topi, and (1/1) impala tested positive for RVFV by serological tests. One blood sample from sheep in Kiboga district tested RVFV positive. All the human cases were exposed through contact or consumption of meat from infected animals.

**Conclusion:**

RVF outbreaks occurred in humans and animals in Kiruhura, Buikwe, Mityana, and Kiboga districts. Human cases were potentially infected through contact with infected animals and their products.

## 1. Introduction

Rift Valley fever (RVF) is a mosquito-borne viral zoonosis that primarily affects animals but can also infect humans. It is a neglected, reemerging disease that causes morbidity in both human and animal populations. The disease is caused by the Rift Valley fever virus (RVFV); an arbovirus in the *Phlebovirus* genus and Phenuiviridae family. Rift Valley fever was first characterized by Daubney et al. in a laboratory in Kenya in 1930 and is now endemic throughout numerous African countries and the Arabian Peninsula [1,2].

Rift Valley fever has a complex lifecycle involving humans, mosquitoes, wild and domesticated animals, and the environment [3]. The virus primarily infects domestic livestock such as cattle, sheep, and goats causing high rates of neonatal mortality and abortion [2]. Rift Valley fever virus has been found in several wild mammals in Africa such as African buffaloes and impalas where it causes mild illness [3–5]. Rift Valley fever virus is transmitted from either mosquitoes or farm animals to humans but person-to-person transmission has not been documented [3]. The primary reservoir and vector for RVFV is the *Aedes* mosquito though can be transmitted by other mosquitoes such as *Anopheles* and *Culex* [6]. *Aedes* mosquitoes can lay infected eggs that can stay in the soil for a long period in dry conditions and hatch during wet months into infected mosquitoes [7]. Therefore, in flooding situations, the infected eggs can be transported to new locations hence spreading the virus to different geographical locations. The infection can also spread due to animal movements introducing infected animals to new territories. The survival of RVFV during inter epizootics is believed to depend on the transovarial transmission of the virus in flood water by *Aedes* mosquitoes [8, 9]. Other mosquitoes in the *Culex* and *Anopheles* genus are thought to be important in the amplification of virus activity during outbreaks [10].

In the animals, transmission is mainly through bites of *Aedes* mosquitoes. However, the disease is mainly acquired in humans through contact with blood, body fluids, or tissue and consumption of raw or undercooked milk or meat from infected animals [2]. This is why persons who interact with animals and their products such as veterinarians, herders, and butchers are a high-risk population for RVF [11].

The disease has an incubation period of 2–6 days in humans and varies in severity; that is, some remain asymptomatic and others might experience mild illness whereas some may have severe disease. Rift Valley fever virus patients may present with fever, generalized weakness, back pain, and dizziness which can clear within 2–7 days. However, 8–10% can develop severe disease characterized with ocular disease, encephalitis, or haemorrhagic fever. About 50% of those that develop severe disease either die or remain with permanent disabilities [11]. RVF outbreaks have mainly been reported in Sub-Saharan Africa; for example, in 2006 through 2007, RVF occurred in East Africa affecting more than 1,000 people with 300 deaths. Uganda reported an RVF outbreak among humans in 2016 and since then over ten sporadic RVF outbreaks have occurred [12]. These outbreaks had a severe negative economic impact; however, limited epidemiological investigations were conducted.

In 2017–2018, the Uganda Ministry of Health (MoH) through the Public Health Emergency Operational Centre (PHEOC) received alerts of suspected viral haemorrhagic fever from Kiruhura, Buikwe, Kiboga, and Mityana districts. Laboratory results from the Uganda Virus Research Institute (UVRI) indicated that human cases were positive for RVFV by PCR. This investigation was therefore conducted to determine the extent of the outbreak, identify exposure factors for transmission, and recommend prevention and control measures.

## 2. Methods

### 2.1. Outbreak Area

**Figure 1:**
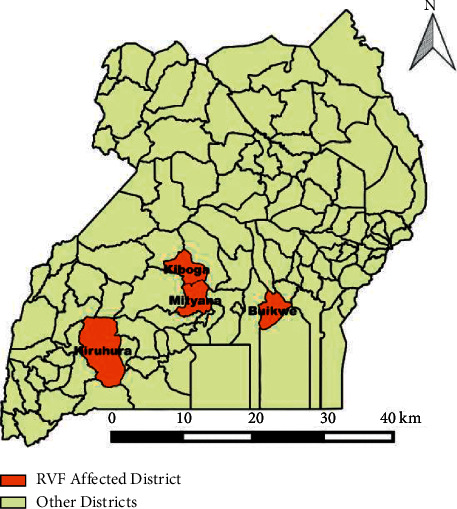
Map of Uganda showing districts affected by Rift Valley fever outbreak, November 2017–January 2018.

Cases were reported from districts of Kiboga, Mityana, Buikwe, and Kiruhura districts in the cattle corridor of Uganda. Mityana district is bordered by Kiboga to the north. The main economic activity carried out in these districts is livestock farming. Three districts (Kiboga, Mityana, and Buikwe) are in the central region while Kiruhura is in the Western region of Uganda (Figure 1).

### 2.2. Case Definition, Finding, and Identification of Exposure Factors for Infection

A suspected case was defined as any person with acute onset of fever (<37.5°C), negative malaria test result, and at least two of the following symptoms: headache, muscle or joint pain, bleeding presentation, and any gastroenteritis symptom (nausea, vomiting, abdominal pain, diarrhoea) in a resident of Kiruhura, Buikwe, Mityana, and Kiboga districts from 1^st^ October 2017 to 30^th^ January 2018. A confirmed case was defined as a suspected case with laboratory confirmation by either detection of RVF nucleic acid by reverse-transcriptase polymerase reaction (RT-PCR) or demonstration of serum IgM or IgG antibodies by ELISA.

Active case finding was conducted in health facilities and communities where cases had been reported in all the districts of Kiruhura, Buikwe, Mityana, and Kiboga. A total of 24 blood samples were collected from human cases.

Standard case investigation forms for viral haemorrhagic fevers were used to collect information from the human cases. In-depth interviews were conducted with human cases that were infected with RVF who included herdsmen and slaughterers/meat handlers to identify exposure factors for RVF infection.

### 2.3. Laboratory Investigations

A total of 362 animal blood samples were collected and tested. Blood samples were purposively collected from farms with livestock where human cases had been confirmed as well as those that reported rampant abortions; death of calves, kids, lambs; unexplained death after a short illness as follows: 146 cattle, 76 goats, and 20 sheep from four selected farms in Kanyaryeru subcounty in Kiruhura district. A convenient sampling method was used to collect 12 blood samples from wildlife (10 zebras, 1 topi, 1 impala) from Lake Mburo National Park (LMNP), Kiruhura district. The location where confirmed human cases were identified was in proximity to LMNP. Wildlife was sampled to find a proxy for exposure among humans. Blood samples from 7 goats, 22 sheep, and 33 cattle from Leprome forest plantation where the confirmed human cases worked in Kiboga district were also collected. All the herds that were sampled had a history of abortions in cattle, goats, and even sheep. A total of 28 animal blood samples were collected from Buikwe district. A total of 18 blood samples were collected from Mityana district (8 goats, 2 sheep, and 8 cattle). We transported the samples to the National Animal Disease Diagnostic and Epidemiology Centre (NADDEC) for testing IgM and IgG antibodies. Blood samples were collected from human cases and were sent to UVRI for testing. PCR was performed on the RNA extractions from the human samples to identify the RVF virus using the TaqMan assay targeting the nonstructural protein-coding region [13]. RVF primers probe were designed from a published Gene Bank and applied using the established methodology. After optimization of the amplification reaction and establishment of the calibration curve with synthetic RNA from the plasmid containing the gene of interest, real-time PCR was assessed with samples containing RVFV from infected cells [14]. Animal specimens were tested for anti-RVFV IgG and IgM using Enzyme-Linked Immunosorbent Assay (ID Screen® Rift Valley fever Competition Multi-species ELISA Montpellier, France) at the NADDEC [15].

### 2.4. Environmental Assessments

Environmental assessments were conducted in all the affected districts. In-depth interviews with herdsmen and slaughterers were conducted to identify any sick or dead animals in the affected areas during the outbreak period. Inquiries were made about the occurrence of sudden rampant abortions in animals, death of young ones (calves, kids, and lambs), and unexplained death of animals after a short illness, floods, above-normal rainfall, increased mosquito populations, overflow of water bodies (to include lakes, rivers, dams, and valley tanks), use of mosquito nets, the vaccination status of the animals, and interaction of wildlife with domestic animals and humans.

## 3. Results

**Figure 2:**
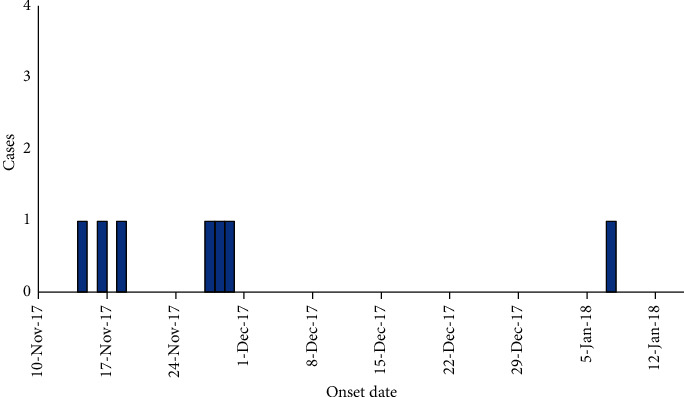
Epidemiologic curve showing all Rift Valley fever cases in Buikwe, Kiboga, Kiruhura, and Mityana, Uganda, November 2017–January 2018.

Of the 24 human blood samples tested, 7 tested positive for RVFV: Kiruhura (4), Buikwe (1), Kiboga (1), and Mityana (1) (Figure 2). Of the seven confirmed human cases, three had both detectable anti-RVF-specific IgM and IgG. One case was positive for only anti-RVF-specific IgG. One case from Kiboga, Mityana, and Buikwe, respectively, had detectable anti-RVF-specific IgM and IgG.

All cases were male with a mean age of 34 years. Three of the seven confirmed human cases died. Most of the human cases presented with signs and symptoms consistent with a viral haemorrhagic fever infection (Table 1). All human cases were classified as health facility alerts who tested positive for RVFV by PCR.

All human cases had a history of contact with cattle likely infected with RVF through slaughtering, butchering, eating, and carrying meat of either sick or dead cattle.

### 3.1. Kiboga District Human Case

This was a 26-year-old male forest worker of Leprome Forest Plantation. Leprome Forest Plantation is in Busakya village, Kajere parish, Kiboga subcounty, Kiboga district, central Uganda. On 16^th^ November 2017, he presented with fever, malaise, and bleeding from the mouth and died shortly after admission. He had a history of contact with sick cattle that died on 19^th^ and 31^st^ October 2017 of unknown cause.

### 3.2. Mityana District Human Case

This was a 51-year-old male peasant farmer. On 16^th^ November 2017, he presented with high-grade fever, bleeding from his nose, and bloody diarrhoea and died on 21^st^ November 2017. It was reported that the case was fond of eating partially cooked meat. In this instance, he had eaten meat from a cow that had recently died of an unknown cause in his neighbourhood.

### 3.3. Buikwe District Human Case

This case was a 60-year-old male butcher from Vvumba village, Busabaga parish, Kawolo subcounty, Lugazi municipality, and Buikwe district. He had animals and herded his own cattle. On 7^th^ January 2018, he developed a high fever and cough. His condition deteriorated between 12^th^ and 14^th^ January 2018 when he started vomiting blood. The patient had slaughtered a sick cow on the 3^rd^ January 2018. It was not inspected by a veterinary doctor before slaughter and the owner had noticed development of signs of an unknown ailment.

### 3.4. Kiruhura District Human Case

There are a total of 4 human cases, all from Rushororo Village in Kanyaryeru subcounty in Kiruhura District, Southwestern Uganda.

**Case 1 .**

A 24-year-old male presented with a high-grade fever and severe headache on 30^th^ November 2017. Epistaxis, hematemesis, extreme weakness, sore throat, hiccups, joint, and muscle pains followed this in 3 days after the onset of symptoms. He had participated in skinning, chopping, and consumption of a 2-week-old calf that died of an unknown illness.

**Case 2 .**

A 39-year-old male had the earliest date of symptom onset. On 18^th^ November 2017, he developed fever and headache, 8 days before the death of the calf. He was found positive with RVF by serological tests (IgG antibodies only) done at UVRI.

**Case 3 .**

A 24-year-old male developed symptoms of fever and headache on 26^th^ November 2017, one day after butchering the dead calf.

**Case 4 .**

**Figure 3:**
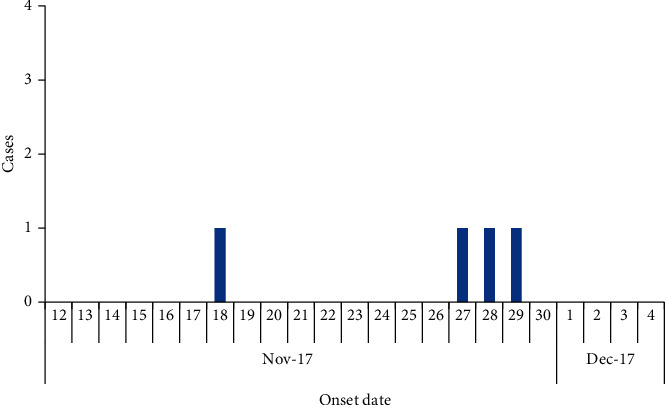
Epi curve showing Rift Valley fever patients, Kiruhura District, November 2017–December 2017.

An 11-year-old male, on 29^th^ November 2017, developed a high-grade fever and headache. He had participated in the slaughtering of the dead calf by carrying offals (Figure 3).

Of the domestic animal samples that were collected from Kiruhura, 44% (64/146) of cattle, 46% (35/76) of goats, and 45% (9/20) of the sheep tested positive for RVFV by serological tests. Among wildlife, 1/10 of zebras, 1/1 of topi, and 1/1 of impala tested positive for RVF by serological tests. All animal blood samples obtained from Kiboga district were negative for RVFV. One of the animal blood samples from Buikwe district tested positive for RVFV.

It was observed that wild animals freely interacted with domestic animals during grazing in Kiruhura district. There were reports of sudden deaths and stillbirths among goats and cattle on various farms in all the affected districts. The presence of wildlife including monkeys and antelopes was also reported in the man-made forest in Kiboga district. Numerous abortions attributed to RVF were reported among animals. All the herds that were sampled had a history of abortions in cattle, goats, and even sheep. It was reported that the *abortus* was usually thrown into the neighboring bushes. There was no evidence of RVF vaccinations in animals in all affected districts. The monthly average amount of rainfall received in all districts throughout 2017 was 1.7 mm (0.0 mm–3.7 mm) did not deviate from the trend of rainfall received the previous year 2016 in which the country registered an RVF outbreak.

## 4. Discussion

The sporadic RVF outbreaks occurred over a period of four months. This was the second occurrence of RVF in humans in Uganda. The first-ever reported RVF in humans was in 2016 in Western Uganda [16]. Evidence of RVF seropositivity was found in human cases in these outbreaks. Our findings suggested that these human cases were exposed through contact or consumption of meat from infected animals. Several studies have suggested that most human infections are the result of direct or indirect contact with blood, secretions, tissue, or organs of infected animals during slaughter, assisting with animal births, or conducting veterinary procedures. For instance, during the 2007 outbreak in Kenya, contact with RVFV-infected animals such as consuming or handling products from sick animals, touching an aborted animal foetus, or being a herdsman was documented as an important risk factor for severe infection [8].

RVF infection was found in both livestock and wildlife. It was observed that wildlife freely interacted with livestock. A case in point is domestic animals were grazing with the wildlife near Lake Mburo National Park (LMNP). This was in conformity with a study conducted in Kenya, which indicated that the presence of RVF virus neutralizing antibodies was demonstrated among wildlife, including African buffalo, black rhino, lesser kudu, impala, African elephant, kongoni, and waterbuck. The same study found a seroprevalence of RVF in wildlife at >15% [17]. This, therefore, indicates that interactions or increased contact among livestock and wildlife may have led to the occurrence of RVF outbreaks in animals in Kiruhura district. According to the results from this outbreak, the impala, zebra, and topi tested positive for RVF suggesting circulation of the virus in the wildlife.

Multiple Rift Valley fever outbreaks have previously been reported in African countries, including South Africa, Somalia, Tanzania, Sudan, Kenya, Senegal, and in the Middle Eastern countries of Saudi Arabia and Yemen [18–20]. For epidemics to occur, three factors must be present, the preexistence or introduction of the virus in the area, the presence of large populations of susceptible ruminants, climatic or environmental conditions that encourage a massive build-up in the vector mosquito population. The latter usually occurs when there are warm conditions. This particular outbreak occurred following a dry season and during an end-of-year festive period, where most probably there was increased importation of animals and these could have been sourced from RVF endemic areas. This was also observed in a risk assessment conducted by Abdo-Salem et al. that highlighted the introduction of RVFV from the horn of Africa to Yemen via the legal trade of small ruminants [21].

In addition, three of the affected districts lie in the cattle corridor of Uganda, so we cannot rule out the fact that livestock markets that seldom occur within the cattle corridor could have facilitated the movement of infected animals to new locations.

Rift Valley fever outbreaks also occur following unusually heavy and persistent rainfalls that cause surface flooding and lead to the hatching of infected *Aedes* spp. mosquito eggs and large numbers of vector mosquitoes. In the RVF outbreak in Sudan, the role of mosquitoes in spreading the outbreak was documented [10]. Since human infections can also occur through bites from infected mosquitoes, most commonly *Aedes* and *Culex* mosquitoes, there is a possibility that human cases might have been exposed through mosquito bites. It was noted that some of the human cases were herdsmen and usually herd animals in bushy places. More so, one of the human cases was working in a forest, which is a habitat for potential vectors including mosquitoes.

While most human cases usually present with relatively mild symptoms and signs, a small percentage of patients develop a much more severe form of the disease. This usually appears as 1 or more of 3 distinct syndromes: ocular (eye) disease, meningoencephalitis, or haemorrhagic fever. Most fatalities occur in patients who develop the haemorrhagic icterus form [22]. From the affected districts, at least one human case died after presenting with haemorrhagic symptoms.

The occurrence of these outbreaks in multiple districts in Uganda suggested RVFV infection distribution to new territories. This scenario was previously seen on a larger scale highlighting seven of the major RVF outbreaks on the African continent indicating spread outside the RVF region in East Africa [23].

Rift Valley fever can be prevented in livestock through vaccination. However, RVF vaccination in animals in Uganda has not yet been practiced because the circulating RVF strain in animals has not been fully investigated and profiled. The vaccine is likely to be administered to some animals that are already infected and viremic, thus facilitating the serial transfer of a wild type virus [3].

Both live and inactivated vaccines have been developed for use in livestock. Two commercially available RVF vaccines on the Ugandan market are live attenuated. Like any other vaccine, these vaccines have their own merits and demerits. For instance, though only one dose of live attenuated vaccine is required to provide long-term immunity, it can result in spontaneous abortion in pregnant animals, teratogenic effects, and the potential creation of a generation of reassortant genotypes [24]. On the other hand, the inactivated vaccine does not have any of these side effects, but multiple doses are needed to provide long-term immunity. Given all these considerations, Uganda must first profile the circulating RVF strain to start vaccination in animals as a strategy to prevent and control the disease.

## 5. Conclusion

RVF outbreaks occurred in humans and animals in Kiruhura, Buikwe, Mityana, and Kiboga districts. Human cases were potentially infected through contact with infected animals and consumption of meat of infected carcasses. Domestic animals were potentially infected through contact with infected wildlife.

### 5.1. Recommendations

The Ministry of Agriculture, Animal Industries and Fisheries (MAAIF) should ensure enforcement of laws towards consumption of sick and dead animals. The communities should be educated about RVF and the risk factors for infection. Any unusual events such as sudden deaths, abortions, and stillbirths observed in farms should be used as proxy indicators for disease in animals including RVF.

Furthermore, efforts to contain the transmission of infections in humans, livestock, and wildlife and ways to reduce exposure of humans to infected animals need to be established. Entomological studies need to be conducted to investigate if mosquitoes in the affected areas are infected with RVFV, thus a possibility of a source of infection to animals.

## Figures and Tables

**Table 1:** Description of symptoms and signs among Rift Valley fever cases, in Buikwe, Kiboga, Kiruhura, and Mityana, Uganda, November 2017–January 2018.

District	Sex	Age	Onset date of symptom	Symptoms/signs								Status
				Fever	Headache	Malaise	Epistasis (vomiting blood)	Bleeding (nose, gums)	Cough	Nausea	Bloody diarrhoea	
Kiboga	M	26	14/11/2017	+	+	+	+					Dead
Mityana	M	51	16/11/2017	+			+	+		+		Dead
Buikwe	M	60	7/11/2018	+			+	+	+			Dead
Kiruhura	M	24	30/11/2017	+	+	+	+				+	Alive
Kiruhura	M	39	18/11/2017	+	+							Alive
Kiruhura	M	24	27/11/2017	+	+	+						Alive
Kiruhura	M	11	29/11/2017	+	+	+						Alive

## Data Availability

Primary data were used to support the findings in this investigation. The dataset used in this investigation is available from the corresponding author upon request.
